# Toward the Identification of a “Renopoietic System”?

**DOI:** 10.1002/stem.140

**Published:** 2009-06-04

**Authors:** Paola Romagnani

**Affiliations:** Excellence Centre for Research, Transfer and High Education DENOthe, University of FlorenceFlorence, Italy

**Keywords:** Renal progenitors, Renal stem cell, CD133, CD24, Kidney, Metanephric mesenchyme

## Abstract

Chronic kidney disease is a leading cause of mortality and morbidity in Western countries and is estimated to affect 11% of the adult population. The possibility of treatment of chronic kidney disease has been severely impaired by our poor knowledge of the regenerative properties of the kidney. Recent results obtained in humans, together with genetic tagging experiments performed in rodents, demonstrated that the epithelial components of the cortical nephron share a unique progenitor, which can generate podocytes as well as tubular cells. Accordingly, lineage tracing experiments demonstrated that bone marrow-derived interstitial or papillary cells are not involved in the repair of injured adult renal epithelium. In addition, assessment of the markers CD24 and CD133 in adult human kidney as well as genetic tagging in rodents allowed us to identify a hierarchical population of renal progenitors arranged in a precise sequence within Bowman's capsule. The results of all of these studies suggest that the kidney contains a “renopoietic system,” with a progenitor localized at the urinary pole of Bowman's capsule, from where it can initiate the replacement and regeneration of glomerular, as well as tubular, epithelial cells. Knowledge of renal progenitor cell biology may enable a better comprehension of the mechanisms of renal repair as well as more effective targeted therapies for acute and chronic kidney diseases.

## INTRODUCTION

**Figure 1:**
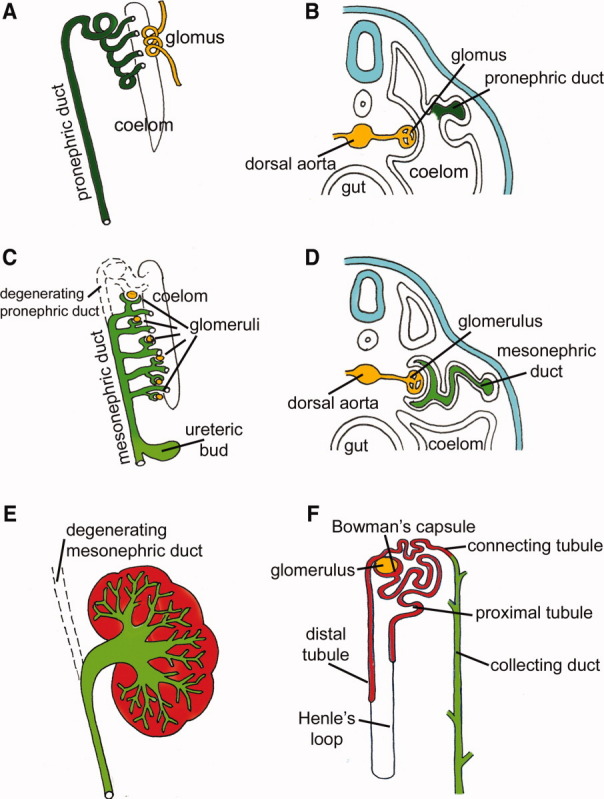
Series of schematic diagrams depicting the morphological events during mammalian kidney development. The mammalian organism forms three excretory organs, all of which are derived from the intermediate mesoderm. **(A, B):** The first and most primitive organ, the pronephros, becomes functional in some fish although it has no obvious function in the mammalian embryo and after a short time it disappears. Longitudinal **(A)** and cross section **(B)** of the pronephros. **(C, D):** The pronephros is replaced by the mesonephros, which is found in high fishes and amphibians, whereas it degenerates and is replaced by the definitive kidney, the metanephros, in mammals. Longitudinal **(C)** and cross section **(D)** of the mesonephros. **(E):** The metanephros generates when the Wolffian duct elongates posteriorly and encounters the metanephric mesenchyma where the ureteric bud emerges. The metanephros and its generation of adult mammalian kidney is shown in a longitudinal section. **(F):** Scheme of a mature nephron and its developmental origin: the glomerulus, proximal tubule, Henle's loop, distal tubule, and connecting tubule derive from the metanephric mesenchyme, whereas the collecting ducts derive from the ureteric bud.

Chronic kidney disease (CKD) is a leading cause of mortality and morbidity in Western countries and is estimated to affect 11% of the adult population [1]. It can progress to end-stage renal disease (ESRD), which has no cure and requires renal replacement therapy, that is, dialysis or renal transplantation. The number of patients with ESRD is growing consistently with rising cumulative costs that are even greater than the direct treatment costs of cancer [1]. The possibility of treatment of CKD has been severely impaired by our poor knowledge of the regenerative properties of the kidney [2]. Indeed, although the resection of an adult kidney does not lead to the regeneration achieved in the liver, the mammalian kidney shares with the majority of organs the ability to repopulate and at least partially repair structures that have sustained some degree of injury [2–4]. In most adult tissues, the process of wounding to replace the damaged or dead cells is maintained through the presence of stem/progenitor cells [5,6]. The discovery of stem cells in adults was introduced by the notion that multipotent progenitors can be found in adult bone marrow and that these cells are responsible for the constant production of blood [7]. These progenitors were thus named as hematopoietic stem cells and are characterized by two main properties, self-renewal and multilineage differentiation potential [7]. It is generally accepted that hematopoietic stem cells are generated during embryonic development and colonize the bone marrow, ultimately giving rise to the adult hematopoietic system, which represents a paradigm in stem cell biology [7]. Learning from the hematopoietic system has helped to identify a pool of tissue-specific, resident, self-renewing, and differentiating progenitors within many analyzed tissues, thus opening the possibility of understanding the regenerative mechanisms of adult tissue. However, the kidney has presented many challenges in the identification and characterization of stem cells [8–14]. Indeed, it was proposed that the cells that elicit kidney repair come from the proliferation of neighboring cells, interstitial or papillary cell transdifferentiation, the recruitment of stem cells from the bone marrow, or the generation of new epithelial cells from an unknown renal stem cell population [2–14]. Taken as a whole, the kidney appears to be extraordinarily complex, but on anatomical analysis this complexity is reducible to fairly simple terms. Each kidney is made up of slightly more than 1 million microscopic units, the nephrons, which are all essentially alike and consist of a filtering bed composed of a capillary tuft, or glomerulus, which drains directly into a long, elaborate tubule [15] (Fig. 1). These million-odd glomerular-tubular units empty into common collecting ducts, which then unify to generate the ureter [15]. Thus, each nephron is functionally and anatomically independent, suggesting that it should harbor its putative stem cell bulk to repair injury. It follows that putative renal stem cells should be functionally characterized by two main properties, self-renewal and multilineage differentiation potential, and might also share some phenotypic properties with hematopoietic stem cells. In addition, nephrons are generated repetitively during kidney organogenesis from a mesenchymal progenitor population [15]. In analogy with the hematopoietic system, a putative stem cell compartment of the kidney may be established during embryonic life. Thus, drawing on understanding of kidney development in search of cells with phenotypic and functional features of multipotent progenitors is crucial to clarifying the possible existence of a “renopoietic system” and to address the mechanisms of renal repair.

## EMBRYONIC RENAL PROGENITOR

**Figure 2:**
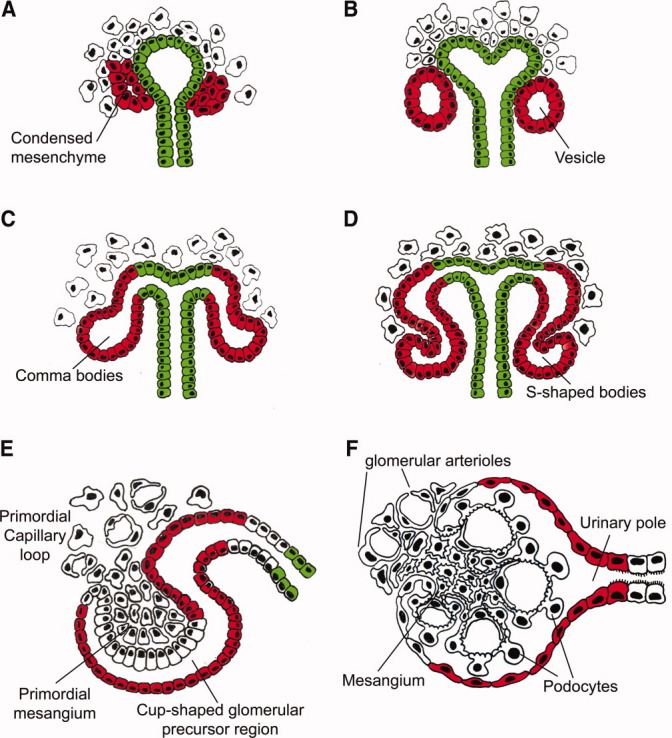
Series of schematic diagrams depicting the morphological events and the localization of CD24^+^CD133^+^ renal progenitors during the different phases of nephron development. Mesenchymal cells near the tips of the branching ureteric bud are induced and differentiate through a series of forms: aggregate **(A),** renal vesicle **(B),** comma-shaped bodies **(C),** and S-shaped bodies **(D)**. Also shown is the developing vasculature within the glomerular cleft of an S-shaped body **(E)** and the glomerulus in a more mature nephron **(F)**. The tubular segments of the mature nephrons empty into the collecting ducts and eventually the ureter. Development proceeds in a radial manner so that older nephrons are centrally located and newer nephrons are added at the periphery as indicated. Cells of the ureteric bud are stained in green. CD24^+^CD133^+^ renal progenitors are stained in red. **(A):** CD24^+^CD133^+^ renal progenitors (red) localize in the condensed mesenchyme but not in the uninduced mesenchyme (white) or in the ureteric bud (green). **(B):** CD24^+^CD133^+^ renal progenitors localize in a primary vesicle but not in the uninduced mesenchyme (white) or in the ureteric bud (green). **(C):** CD24^+^CD133^+^ renal progenitors localize in the comma bodies but not in the uninduced mesenchyme (white) or in the ureteric bud (green). **(D):** CD24^+^CD133^+^ renal progenitors localize in the S-shaped body, in the proximal loop, as well as in the distal loop, but not in the uninduced mesenchyme (white) or in the ureteric bud (green). **(E):** CD24^+^CD133^+^ renal progenitors (red) localize in an S-shaped body after colonization by primordial capillaries and mesangium. **(F):** In maturing glomeruli, CD24^+^CD133^+^ renal progenitors (red) selectively persist as a subset of cells of the Bowman's capsule localized opposite to the vascular pole.

When we talk about kidney formation, we should be aware that the mammalian organism forms three excretory organs, all of which are derived from the intermediate mesoderm (Fig. 1) [15,16]. The first and most primitive organ, called the pronephros, forms in a rudimentary way and becomes functional in some fish, whereas it has no obvious function in the mammalian embryo (Fig. 1A, 1B) [15,16]. After a short time, it disappears and is replaced by the second organ, the mesonephros (Fig. 1C, 1D), which is found in higher fish and amphibians, where it also functions as an hemopoietic organ and is composed of nephrons surrounded by hematopoietic and lymphoid tissue dispersed throughout the organ [15–17]. This strict relationship between the kidney and the bone marrow during development is explained by the observation that the mesonephros represents a relevant portion of an axial, mesodermally derived region of the embryo, the aorta-gonad-mesonephros [18]. The aorta-gonad-mesonephros serves as a major niche for development of hematopoietic stem cells as well as of a variety of mesenchymal progenitors, including the mesenchymal aggregates that generate the third kidney design, the metanephros, which represents the adult mammalian kidney in birds and mammals (Fig. 1E) [18]. Induction of the mature kidney occurs through reciprocal interactions between an epithelial component, the Wolffian duct, and the metanephric mesenchyme. During the induction process, cells of the metanephric mesenchyme condense around the tips of the growing bud; then they undergo a burst of proliferation and convert to an epithelial cell type (Fig. 2). These early epithelial cells form a spherical cyst called the renal vesicle [15,16] (Fig. 2B). The renal vesicle then undergoes a series of invaginations and elongations to generate the comma-shaped (Fig. 2C) and then the S-shaped bodies (Fig. 2D). At this stage, the proximal end of the S-shaped body becomes invaded by blood vessels, differentiates into podocytes and parietal epithelial cells, and then generates the glomerular tuft [15,16] (Fig. 2E, 2F). Simultaneously, the middle and the distal segments of the S-shaped body that had remained in contact with the ureteric bud epithelium fuse to form a single, continuous epithelial tube and begin to express proteins that are characteristic of tubular epithelia [15,16]. Concurrent with these events the growth and repeated branching of the ureteric bud form the system of the collecting ducts that join with the nephron and empty into the ureter. These inductive steps are reiterated throughout the growing kidney so that older nephrons are located in the center and newer nephrons are added at the periphery. In humans the process continues until it stops immediately before birth. Mostly based on clonal analysis in vitro and ex vivo, several researchers previously suggested that individual nephron progenitors are multipotent in their capacity to generate distinct regions of the nephron [19,20] and that they constitute a common embryonic progenitor population for tubular and glomerular epithelial cells. However, which cells within this pool represent renal progenitors and how multiple nephron lineages form during this protracted developmental process was unclear. Very recently, studies performed in transgenic rodents for the homeodomain transcriptional regulator Six2 have demonstrated the existence of a multipotent nephron progenitor population in the cap mesenchyme [21]. Indeed, pulse labeling of *Six2*-expressing nephron progenitors at the onset of kidney development suggested that the *Six2*-expressing population is maintained by self-renewal and is multipotent, generating the multiple domains of the whole cortical nephron [21]. Accordingly, descendants of a Six2^+^ cell can be found within molecularly distinct compartments of a single nephron: podocytes, proximal and distal tubule structures, and the connecting tubule, demonstrating that a single multipotent progenitor is the source of both the glomerular and tubular epithelial cells that constitute the adult cortical nephron [21]. Although lineage tracing studies in rodents definitively proved the existence of a common renal embryonic progenitor for glomerular and tubular epithelial cells, these strategies cannot be applied in humans. However, by using the application of a combination of markers such as CD24, a surface molecule that has been used to identify different types of human stem cells [22–24], CD133 (a marker of hematopoietic and other types of adult tissue stem cells) [24,25], the Polycomb group protein Bmi-1 (a transcription factor that is critical for maintenance of stem cell self renewal) [26,27], and the stem cell-specific transcription factors Oct-4 [28], we identified a subset of cells in embryonic human kidneys that exhibit stem cell phenotypic and functional features [29]. Indeed, surface expression of CD133 and CD24 allowed us to recover a subset of cells in the primordial nephron that displayed self-renewal and multidifferentiation potential [29] (Fig. 2). When injected into mice with acute renal failure, CD24^+^CD133^+^ progenitors purified from embryonic tissues regenerate cells of different portions of the nephron, reduce tissue necrosis and fibrosis, and improve renal function [29]. CD24^+^CD133^+^ progenitors are located in condensed mesenchyme-derived primordial structures (Fig. 2A), primary vesicles (Fig. 2B), comma-shaped bodies (Fig. 2C), and S-shaped bodies (Fig. 2D). In S-shaped bodies, CD24^+^CD133^+^ progenitors are located in the proximal loop, which will give rise to both podocytes and Bowman's capsule of the primitive glomerulus, as well as in the distal loop (Fig. 2D); however, when a primitive vascular tuft is evident within the cup-shaped glomerular precursor region, CD24^+^CD133^+^ progenitors are detected only in parietal epithelial cells of Bowman's capsule (Fig. 2E). In maturing glomeruli, CD24^+^CD133^+^ progenitors are selectively localized at the urinary pole of Bowman's capsule (Fig. 2F). Accordingly, CD24^+^CD133^+^ progenitors are enriched in the kidneys at 8-9 weeks of gestation, when the developing kidney consists mostly of immature metanephric mesenchyme-derived structures, whereas they substantially decrease during 10-14 weeks of gestation when terminally differentiated nephrons appear, representing <2% of whole renal cells in mature kidneys [29]. Taken altogether, these data suggest that a single multipotent progenitor is the source of both glomerular and tubular epithelial cells that constitute the cortical nephron in mammalian kidneys during development.

## ADULT RENAL PROGENITOR

**Figure 3:**
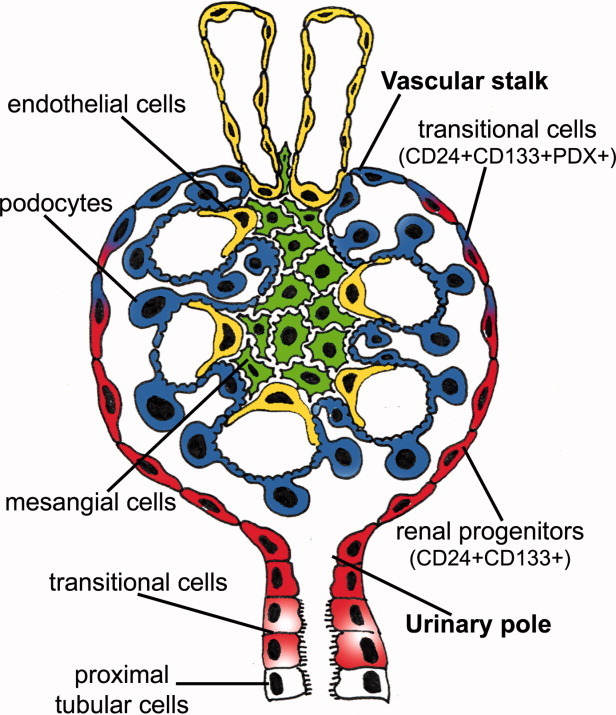
Hierarchical distribution of renal progenitors in adult human glomeruli. CD24^+^CD133^+^ renal progenitors (red) are localized at the urinary pole and are in close contiguity with podocytes (blue) at one extremity (the vascular stalk) and with tubular renal cells (white) at the other extremity. A transitional cell population (CD24^+^CD133^+^PDX^+^, red/blue) displays features of either renal progenitors (red) or podocytes (blue) and localizes between the urinary pole and the vascular pole. At the vascular stalk of the glomerulus, the transitional cells are localized in close continuity with cells that lack progenitor markers but exhibit the podocyte markers and the phenotypic features of differentiated podocytes (blue). Endothelial cells: yellow; mesangial cells: green; podocytes: blue; proximal tubular cells: white; renal progenitors: red.

The observation that nephrons are generated repetitively during kidney organogenesis from a single multipotent progenitor population suggests that small numbers of renal progenitors could exist also in the adult and contribute to kidney homeostasis and repair after injury. In addition, phylogenetic observations suggest the existence of renal progenitors in adult kidneys. Indeed, in response to acute renal injury, the adult fish kidney exhibits a nephron neogenic response with de novo nephron development [17]. Although a similar neogenic response is not observed in mammals, the new nephrons that form in fish after injury follow the same pattern of development as is observed during nephrogenesis in developing mammalian kidneys [15–17]. Specifically, basophilic clusters of cells adjacent to collecting ducts form renal vesicles and S-shaped tubules, and the tubular outgrowths then fuse with the collecting ducts. Glomerular development results in glomeruli with vascular tufts, parietal and visceral epithelia, and a clear Bowman's space [17]. The nephrons develop over a period of 2-4 weeks after induction of injury, suggesting that development of new nephrons requires long periods of time in adult kidneys [17]. This neogenic response provides a unique model for studying developing nephrons in an adult vertebrate organism and suggests that renal progenitors could persist in adult kidneys also after mammalian development. Drawing on developmental processes, Humphreys et al. [30] have performed lineage tracing experiments using transgenic mice to mark cells derived from the renal embryonic progenitors in adult kidneys. In this model, the Six2 promoter drives a fusion protein of green fluorescent protein (GFP) and Cre recombinase. GFPCre is expressed transiently in renal epithelial precursors during the developmental period of active nephrogenesis [30]. No GFPCre expression is present in the adult, and no expression is observed after injury. When Six2-GFPCre mice are crossed with a floxed STOP reporter strain, Cre-dependent removal of the stop sequence in progeny leads to constitutive and heritable expression of a marker gene such that all mesenchyme-derived renal epithelial cells, from Bowman's capsule to the junction of the connecting segment and collecting duct, are heritably labeled [30]. In contrast, the entire interstitial compartment and the collecting ducts are unlabeled. After the induction of acute kidney injury, the repair of injured nephrons was accomplished by intrinsic cells localized within the GFP-labeled cortical nephron, suggesting that repair of injured nephrons is predominantly accomplished only by one or more population(s) of resident epithelial renal cells that represent the direct progeny of the *Six2*-expressing population renal embryonic progenitor. Of note, these results also demonstrate that the contribution of any extratubular renal stem/progenitor cell to repopulation of the tubular epithelium is irrelevant. This is noteworthy, because previous studies suggested that progenitor cells with regenerating potential for renal epithelium could be located in the renal interstitium or among cells of the collecting ducts [8–10]. However, this does not rule out a potential role for interstitial cells in the repair process in response to damage, possibly through secretion of growth factors or cytokines that induce the migration/proliferation of tubular epithelial cells [31]. The absence of label dilution after injury and repair also definitively demonstrates that bone marrow-derived cells do not directly contribute to renal repair, a finding that had already been suggested also by other studies [32,33]. A possible relationship between the embryonic renal progenitor and a putative adult progenitor is also suggested by studies in humans. Indeed, assessment of the CD24 and CD133 markers in adult human kidney allowed us to identify a population of renal progenitors selectively localized at the urinary pole of Bowman's capsule [34] (Fig. 3). The conclusion that CD24^+^CD133^+^ cells in the adult human kidney represent a residual population directly derived from the CD24^+^CD133^+^ embryonic kidney progenitor population cannot be definitively established without lineage tracking, although the similarity of location and the analysis of phenotypic and functional features suggest a strict relationship between the human CD24^+^CD133^+^ progenitors isolated from embryonic kidneys and those isolated from adult kidneys. Accordingly, CD24^+^CD133^+^ progenitors isolated from adult human kidneys also exhibited self-renewal potential, regenerated tubular structures in different portions of the nephron, and reduced the morphologic and functional kidney damage in mice affected by acute renal failure, suggesting that these cells can participate in tubular regeneration in adult human kidneys [3,12,34]. Interestingly several previous studies demonstrated that the proximal tubule arises at a variety of angles from Bowman's capsule and that at least one part of the tubuloglomerular junction has an area of intermediate appearance, with prominent microvilli on parietal cells in humans, mammals, and fish (Fig. 3). These findings suggest that parietal epithelium may be able to change to tubular and that this change may occur particularly as the kidney grows, during severe renal disorders [35–39], or during aging [40]. However, in most cases repair of epithelial cells does not depend on cells generated from multipotent progenitors but directly derives from the migration of epithelial cells from the neighboring epithelia, as reported previously also for the skin [41,42]. Several studies suggested that the tubular epithelium can be self-renewing after acute kidney injury and that differentiated tubular cells proliferate and migrate to replace the neighboring dead cells [32,33,43]. Interestingly, Vogetseder et al. [44,45] proposed that the rapid proliferative response of the tubular epithelium to injury stems from the fact that the tubular epithelium is resting in the G_1_ phase rather than G_0_ and, thus, is primed to enter the cell cycle if disturbed. The fact that a large fraction of the cells are in the G_1_ phase of the cell cycle, ensures a rapid proliferative response when needed. Thus, renal stem/progenitor cells might contribute to tubular epithelium repair, but this contribution probably occurs only when a renal tubular injury cannot be spontaneously repaired through the migration of neighboring unwounded tubular cells.

However, most renal pathological conditions that ultimately lead to ESRD originate within the glomerulus, and it has now been established that depletion of podocytes, the visceral epithelium of the capillary tuft, is central in initiation of glomerulosclerosis, which is the common final pathway of all glomerular diseases leading to ESRD [46–48]. In glomerular diseases such as diabetic nephropathy, glomerulonephritis, or preeclampsia, significant numbers of podocytes are lost as a result of apoptosis, necrosis, or excretion of living cells into the urine. Unlike tubular cells, podocytes are postmitotic cells that cannot undergo complete cell division and are therefore unable to regenerate themselves [48,49]. Interestingly, CD24^+^CD133^+^ progenitors are physically located within Bowman's capsule, the only place in the kidney that appears to be contiguous with both tubular cells and glomerular podocytes [12], suggesting that CD24^+^CD133^+^ progenitors might be responsible for podocyte regeneration after injury. Accordingly, previous studies have suggested the existence of transitional cells exhibiting a mixed phenotype between the parietal epithelial cells and the podocyte at the vascular pole of the glomerulus [50–52] (Fig. 3). In addition, CD24^+^CD133^+^ progenitors represent common progenitors of tubular cells and podocytes during renal development [29]. Very recently, we demonstrated that CD24^+^CD133^+^ cells consist of a hierarchical population of progenitors that are arranged in a precise sequence within Bowman's capsule and exhibit heterogeneous potential for differentiation and regeneration [53]. Cells localized to the urinary pole that expressed CD133 and CD24, but not podocalyxin (PDX) or other podocyte markers (CD24^+^CD133^+^PDX^−^ cells), could regenerate both tubular cells and podocytes (Fig. 3). In contrast, cells localized between the urinary pole and vascular pole that expressed both progenitor and podocytes markers (CD24^+^CD133^+^PDX^+^) could regenerate only podocytes [53] (Fig. 3). Finally, cells localized to the vascular pole did not exhibit progenitor markers but displayed phenotypic features of differentiated podocytes (CD24^−^CD133^−^PDX^+^ cells) [53] (Fig. 3). These findings in humans were also confirmed in a parallel study performed in rodents by Appel et al. [54], who also demonstrated that transitional cells with morphological and immunohistochemical features of both parietal epithelial cells and podocytes could be detected at the glomerular vascular stalk. More importantly, using an elegant model of genetic tagging of parietal epithelial cells in a triple-transgenic doxycycline-inducible mouse line, the same authors unequivocally demonstrated that podocytes are recruited from parietal epithelial cells, which proliferate and differentiate from the urinary to the vascular stalk, generating novel podocytes. Thus, these findings suggest that within the glomerulus a gradient exists, whereby podocytes at the glomerular tip would represent the “oldest” podocytes, which in turn might be most susceptible to damage. In summary, various lines of evidence demonstrate that podocytes can be recruited from a progenitor population localized at the urinary pole of Bowman's capsule. These findings explain how the growing glomerulus is covered with podocytes despite their inability to undergo cell division.

## TOWARD THE IDENTIFICATION OF A RENOPOIETIC SYSTEM

Remission of disease and regression of renal lesions are widely observed in experimental animals and even in humans [4]. However, the repair of injured renal tissue in mammals is not sustained by the generation of new nephrons and frequently leads to a nonfunctioning mass of fibrotic tissue [4]. In contrast, early in gestation, injured fetal tissues can be completely recreated, without fibrosis, in a process resembling regeneration [15,16]. In addition, in response to acute damage, some fish display an effective neo-nephrogenic potential in adulthood [16] whereby new nephrons form from a residual progenitor pool following a sequence that strictly resembles the development of mammalian nephrons [15–17]. Although mammals have retained much of the molecular machinery used by organisms such as fish, their regenerative potential is only limited. So how does the adult kidney replenish cells lost through damage or aging? Recent results demonstrate that, during development, mammalian nephrons derive from a single multipotent progenitor that generates glomerular as well as tubular epithelial cells and that cells with identical markers and properties also exist in adult mammalian kidneys [3,12,21,29,30,33,53,54]. Adult renal progenitors are localized at the urinary pole of Bowman's capsule, the only place of the nephron from where they can initiate the replacement and regeneration of glomerular, as well as tubular, epithelial cells [3,12,33,53,54]. Thus, converging evidence suggests that the kidney contains a renopoietic system and that the urinary pole of Bowman's capsule may represent a stem cell niche, which is a specific site in adult tissues where stem cells reside [5,55]. Accordingly, embryonic stem cells, once differentiated toward renal tubular cells, migrated to the urinary pole of Bowman's capsule after injection into developing kidneys [56], which is a selective property of stem cell niches [5,55].

The existence of a renal progenitor system in adult mammalian kidneys raises the question of why the kidney's regenerative capacity manifests only in some cases and, why, given the high structural and molecular similarities, it does not lead to generation of new nephrons as occurs in human fetuses or in fish. As also suggested for other types of adult epithelia, this probably results from the rapid interposition of fibrotic tissue, which confers a survival advantage by preventing infection but impairs subsequent tissue regeneration [14,57,58]. The switch between regeneration and fibrotic healing is mostly controlled by the immune responses, explaining why human fetuses, as well as fish, which have immature or primordial immune systems, heal without scarring, and can generate new nephrons [14,59]. Hence, the discovery of renal progenitors to encourage regeneration to promote functional repair of tubular and, more importantly, glomerular injury may be possible. In addition, knowledge of renal progenitor cells biology may help to unlock latent regenerative pathways in human kidney [60], which would change medical practice as much as the introduction of transplantation did in the 20th century.

## References

[1] Szczech LA, Lazar IL (2004). Projecting the United States ESRD population: issues regarding treatment of patients with ESRD. Kidney Int Suppl.

[2] Little MH (2006). Regrow or repair: potential regenerative therapies for the kidney. J Am Soc Nephrol.

[3] Sagrinati C, Ronconi E, Lazzeri E (2008). Stem-cell approaches for kidney repair: choosing the right cells. Trends Mol Med.

[4] Remuzzi G, Benigni A, Remuzzi A (2006). Mechanisms of progression and regression of renal lesions of chronic nephropathies and diabetes. J Clin Invest.

[5] Blanpain C, Horsley V, Fuchs E (2007). Epithelial stem cells: turning over new leaves. Cell.

[6] Gurtner GC, Werner S, Barrandon Y (2008). Wound repair and regeneration. Nature.

[7] Cumano A, Godin I (2007). Ontogeny of the hematopoietic system. Annu Rev Immunol.

[8] Oliver JA, Maarouf O, Cheema FH (2004). The renal papilla is a niche for adult kidney stem cells. J Clin Invest.

[9] Dekel B, Zangi L, Shezen E (2006). Isolation and characterization of nontubular Sca-1^+^Lin^−^ multipotent stem/progenitor cells from adult mouse kidney. J Am Soc Nephrol.

[10] Bussolati B, Bruno S, Grange C (2005). Isolation of renal progenitor cells from adult human kidney. Am J Pathol.

[11] Gupta S, Verfaillie C, Chmielewski D (2006). Isolation and characterization of kidney-derived stem cells. J Am Soc Nephrol.

[12] Challen GA, Bertoncello I, Deane JA (2006). Kidney side population reveals multilineage potential and renal functional capacity but also cellular heterogeneity. J Am Soc Nephrol.

[13] Maeshima A, Yamashita S, Nojima Y (2003). Identification of renal progenitor-like tubular cells that participate in the regeneration processes of the kidney. J Am Soc Nephrol.

[14] Sagrinati C, Netti GS, Mazzinghi B (2006). Isolation and characterization of multipotent progenitor cells from the Bowman's capsule of adult human kidneys. J Am Soc Nephrol.

[15] Dressler GR (2006). The cellular basis of kidney development. Annu Rev Cell Dev Biol.

[16] Schedl A (2007). Renal abnormalities and their developmental origin. Nat Rev Genet.

[17] Reimschüssel R (2001). A fish model of renal regeneration and development. ILAR J.

[18] Sainio K, Raatikainen-Ahokas A (1999). Mesonephric kidney—a stem cell factory?. Int J Dev Biol.

[19] Herzlinger D, Koseki C, Mikawa T (1992). Metanephric mesenchyme contains multipotent stem cells whose fate is restricted after induction. Development.

[20] Osafune K, Takasato M, Kispert A (2006). Identification of multipotent progenitors in the embryonic mouse kidney by a novel colony-forming assay. Development.

[21] Kobayashi A, Valerius MT, Mugford JW (2008). Six2 defines and regulates a multipotent self-renewing nephron progenitor population throughout mammalian kidney development. Cell Stem Cell.

[22] Shackleton M, Vaillant F, Simpson KJ (2006). Generation of a functional mammary gland from a single stem cell. Nature.

[23] Kubota H, Avarbock MR, Brinster RL (2003). Spermatogonial stem cells share some, but not all, phenotypic and functional characteristics with other stem cells. Proc Natl Acad Sci USA.

[24] Coskun V, Wu H, Blanchi B (2008). CD133^+^ neural stem cells in the ependyma of mammalian postnatal forebrain. Proc Natl Acad Sci USA.

[25] Kania G, Corbeil D, Fuchs J (2005). Somatic stem cell marker prominin-1/CD133 is expressed in embryonic stem cell-derived progenitors. Stem Cells.

[26] Rajasekhar VK, Begemann M (2007). Roles of Polycomb group proteins in development and disease: a stem cell perspective. Stem Cells.

[27] Kozakowski N, Soleiman A, Pammer J (2008). BMI-1 expression is inversely correlated with the grading of renal clear cell carcinoma. Pathol Oncol Res.

[28] Pesce M, Schöler HR (2001). Oct-4: gatekeeper in the beginnings of mammalian development. Stem Cells.

[29] Lazzeri E, Crescioli C, Ronconi E (2007). Regenerative potential of embryonic renal multipotent progenitors in acute renal failure. J Am Soc Nephrol.

[30] Humphreys BD, Valerius MT, Kobayashi A (2008). Intrinsic epithelial cells repair the kidney after injury. Cell Stem Cell.

[31] Kaissling B, Le Hir M (2008). The renal cortical interstitium: morphological and functional aspects. Histochem Cell Biol.

[32] Duffield JS, Park KM, Hsiao LL (2005). Restoration of tubular epithelial cells during repair of the postischemic kidney occurs independently of bone marrow-derived stem cells. J Clin Invest.

[33] Lin F, Moran A, Igarashi P (2005). Intrarenal cells, not bone marrow-derived cells, are the major source for regeneration in postischemic kidney. J Clin Invest.

[34] Mazzinghi B, Ronconi E, Lazzeri E (2008). Essential but differential role for CXCR4 and CXCR7 in the therapeutic homing of human renal progenitor cells. J Exp Med.

[35] Finckh ES, Joske RA (1954). The occurrence of columnar epithelium in Bowman's capsule. J Path Bact.

[36] Nachman RL (1962). Metaplasia of parietal capsular epithelium of renal glomerulus. Arch Pathol.

[37] Kanel GC, Peters RL (1984). Glomerular tubular reflux—a morphologic renal lesion associated with the hepatorenal syndrome. Hepatology.

[38] Valdes AJ, Zhang JM (1987). Intraglomerular tubular epithelial cells: a marker of glomerular hematuria. Arch Pathol Lab Med.

[39] Andrews PM (1981). The presence of proximal tubulelike cells in the kidney parietal epithelium in response to unilateral nephrectomy. Anat Rec.

[40] Castelletto L, Goya RG (1990). Sex-related incidence of tubular metaplasia in Bowman's capsule of aging rats. Virchows Arch B Cell Pathol Incl Mol Pathol.

[41] Ito M, Liu Y, Yang Z (2005). Stem cells in the hair follicle bulge contribute to wound repair but not to homeostasis of the epidermis. Nat Med.

[42] Levy V, Lindon C, Harfe BD (2005). Distinct stem cell populations regenerate the follicle and interfollicular epidermis. Dev Cell.

[43] Liu KD, Brakeman PR (2008). Renal repair and recovery. Crit Care Med.

[44] Vogetseder A, Picard N, Gaspert A (2008). Proliferation capacity of the renal proximal tubule involves the bulk of differentiated epithelial cells. Am J Physiol Cell Physiol.

[45] Vogetseder A, Palan T, Bacic D (2007). Proximal tubular epithelial cells are generated by division of differentiated cells in the healthy kidney. Am J Physiol Cell Physiol.

[46] Kriz W, Gretz N, Lemley KV (1998). Progression of glomerular diseases: is the podocyte the culprit?. Kidney Int.

[47] Wharram BL, Goyal M, Wiggins JE (2005). Podocyte depletion causes glomerulosclerosis: diphtheria toxin-induced podocyte depletion in rats expressing human diphtheria toxin receptor transgene. J Am Soc Nephrol.

[48] Wiggins RC (2007). The spectrum of podocytopathies: a unifying view of glomerular diseases. Kidney Int.

[49] Marshall CB, Shankland SJ (2006). Cell cycle and glomerular disease: a minireview. Nephron Exp Nephrol.

[50] Kelly G, Downie I, Gardiner DS (1990). The peripolar cell: a distinctive cell type in the mammalian glomerulus. Morphological evidence from a study of sheep. J Anat.

[51] Thumwood CM, McCausland J, Alcorn D (1993). Scanning and transmission electron-microscopic study of peripolar cells in the newborn lamb kidney. Cell Tissue Res.

[52] Bariety J, Mandet C, Hill GS (2006). Parietal podocytes in normal human glomeruli. J Am Soc Nephrol.

[53] Ronconi E, Sagrinati C, Angelotti ML (2009). Regeneration of glomerular podocytes by human renal progenitors. J Am Soc Nephrol.

[54] Appel D, Kershaw DB, Smeets B (2009). Recruitment of podocytes from glomerular parietal epithelial cells. J Am Soc Nephrol.

[55] Rizvi AZ, Wong MH (2005). Stem cell niche: there's no place like home. Stem Cells.

[56] Kim D, Dressler GR (2005). Nephrogenic factors promote differentiation of mouse embryonic stem cells into renal epithelia. J Am Soc Nephrol.

[57] Colwell AS, Longaker MT, Lorenz HP (2003). Fetal wound healing. Front Biosci.

[58] Gurtner GC, Callaghan MJ, Longaker MT (2007). Progress and potential for regenerative medicine. Annu Rev Med.

[59] Mescher AL, Neff AW (2005). Regenerative capacity and the developing immune system. Adv Biochem Eng Biotechnol.

[60] Romagnani P, Lasagni L (2007). Pharmacological modulation of stem cell function. Curr Med Chem.

